# Sputum microbe community alterations induced by long-term inhaled corticosteroid use are associated with airway function in chronic obstructive pulmonary disease patients based on metagenomic next-generation sequencing (mNGS)

**DOI:** 10.3389/fphar.2024.1323613

**Published:** 2024-06-10

**Authors:** Yuanyi Yue, Baohui Zhang, Zhong He, Yuling Zheng, Xueqing Wang, Qiang Zhang

**Affiliations:** ^1^ Department of Gastroenterology, Shengjing Hospital of China Medical University, Shenyang, China; ^2^ Department of Neurobiology, China Medical University, Shenyang, China; ^3^ Journal Center, China Medical University, Shenyang, China; ^4^ Department of Pulmonary and Critical Care Medicine, Shengjing Hospital of China Medical University, Shenyang, China; ^5^ Genoxor Medical Science and Technology Inc., Taizhou, China

**Keywords:** chronic obstructive pulmonary disease, inhaled corticosteroids, sputum microbiota, airway function, metagenomic next-generation sequencing

## Abstract

**Objective:** Inhaled corticosteroids (ICS) are widely used in chronic obstructive pulmonary disease (COPD) patients as a treatment option. However, ICS may also increase the risk of pneumonia and alter the composition of airway microbiota. In clinical application, the overuse of ICS exists pervasively and may potentially lead to adverse effects. Whether the long-term use of ICS confers enough benefit to COPD patients to justify its use so far remains unknown. Therefore, this study employed a single-center retrospective cohort study to compare alterations in airway function and the sputum microbial community structure between COPD patients who had undergone either long-term or short-term treatment with ICS.

**Methods:** Sixty stable COPD patients who had used ICS were recruited and classified into the long-term use group (more than 3 months) and short-term use group (less than 3 months). The demographic features and clinical information of the subjects were investigated and their sputum samples were collected and subjected to metagenomic next-generation sequencing (mNGS).

**Results:** The study found that compared with short-term ICS use, long-term ICS use did not further improve the clinical airway function, decrease the number of acute exacerbations, or decrease hospital readmission. In terms of sputum microbiota, the long-term use of ICS significantly altered the beta diversity of the microbial community structure (*p* < 0.05) and the top three phyla differed between the two groups. At the genus level, long-term ICS induced higher relative abundances of *Abiotrophia*, *Schaalia*, *Granulicatella*, *Mogibacterium*, *Sphingobium*, and *Paraeggerthella* compared to short-term ICS use. Additionally, alpha diversity was positively associated with clinical airway indicators (pre-bronchodilatory FEV1 and pre-bronchodilatory FVC) in the long-term ICS group. The relative abundances of *Rothia*, *Granulicatella*, *Schaalia*, and *Mogibacterium* genera had positive correlations with the eosinophil % (of all white blood cells).

**Conclusion:** This study reveals the effect of long-term and short-term ICS use on sputum microbiota among COPD patients and provides a reference for the appropriate application of clinical ICS treatment in COPD patients.

## 1 Introduction

Inhaled corticosteroids (ICS) are one of the most commonly prescribed medications in chronic obstructive pulmonary disease (COPD) patients. Treatment with ICS prescriptions has been reported to provide sustained anti-inflammatory effects (Falk et al., 2008; Zhai et al., 2017), especially for COPD patients who carry the risk of acute exacerbation. The combination use of ICS, long-acting β-agonist (LABA), and long-acting muscarinic antagonists (LAMA) as a triple therapy is commonly used, and this regimen has been reported to improve symptoms and reduce the rate of mortality (Cazzola et al., 2018; Lipson et al., 2018). Regrettably, ICS prescriptions still have some unexplained drawbacks, such as conferring a possible increased risk of lower respiratory tract infections and pneumonia in COPD patients (Crim et al., 2009; Lee et al., 2013). The most recent Global Initiative for Chronic Obstructive Lung Disease (GOLD) report (2023) recommends that ICS should only be administered in COPD patients who have a high risk of frequent exacerbations and on the basis of consideration of blood eosinophil indicators (GOLD, 2023). However, evidence from real-world studies shows that the phenomenon of ICS overuse is pervasive, sometimes with triple therapy (its conjunction with LABA and LAMA) having been used as a common maintenance treatment for COPD patients (Lucas et al., 2008; Parikh et al., 2020). To date, whether the long-term use of ICS triple therapy brings enough benefit to COPD patients, and the relevant mechanisms of its action, remain poorly understood.

Mouth and airway microbial communities are essential components of the human microbiota. As a previous study reported, the community structure and signature of airway microbiota play an essential role in the development and outcome of COPD(Budden et al., 2019). Bacterial colonization is one of the common causes of the condition known as acute exacerbation of COPD (AECOPD) (Sethi et al., 2006), while lower alpha diversity of microflora in the respiratory tract is related to a higher risk of AECOPD(Pragman et al., 2018). At the phylum level, Firmicutes, Bacteroidetes, and Actinobacteria phyla are dominant in healthy adults, while Proteobacteria is the most abundant microbe in stable COPD and AECOPD patients (Haldar et al., 2020). In addition, findings from cohort studies suggest that there is lower relative abundance of *Veillonella* and higher relative abundance of *Staphylococcus* in non-surviving AECOPD patients within 1 year, while differences were also found in the alpha and beta diversity of the sputum microbiome between survivors and non-survivors (Leitao Filho et al., 2019). Likewise, as important processes underlying the development of COPD, oxidative stress (Zhao et al., 2022) and inflammation have been associated with the imbalance of microorganisms (Sekine et al., 2012; Zhang et al., 2021). Acute bacterial airway infection and bacterial colonization can trigger an acute inflammatory response and promote the acute exacerbation of COPD (Sethi and Murphy, 2008). As Ramsheh et al. demonstrated, a lower relative abundance of *Prevotella* and a greater relative abundance of *Moraxella* were associated with the upregulation of proinflammatory responses and the downregulation of epithelial defense gene expression (Ramsheh et al., 2021). *Moraxella catarrhalis* and non-typeable *Haemophilus influenzae* frequently exacerbate COPD via inflammatory oxidative stress (Nicchi et al., 2022).

Likewise, the use of ICS can alter the airway microenvironment and further affect the structure of the microbial community residing within it. Based on 16S rRNA sequence analyses, ICS has been associated with an increase in sputum bacterial load and can modulate the beta diversity of oral and sputum microbiota (Contoli et al., 2017; Melo-Dias et al., 2023). Treatment with ICS/LABA dual therapy increased the relative abundance of Firmicutes and decreased the relative abundance of Proteobacteria compared to treatment with LABA alone in COPD patients (Contoli et al., 2017). The ICS dose could also be a risk factor for bacterial infection. AECOPD patients administered a higher dose of ICS were found to have a higher rate of pathogenic bacterial colonization (Ko et al., 2008) and increased susceptibility to *Pseudomonas aeruginosa* infection compared with those on a low or medium dose of ICS (Shafiek et al., 2021). However, whether the regular use of ICS prescriptions (the duration of ICS use) affects the structure and diversity of sputum microbiota has been unclear.

Therefore, this study enrolled COPD patients with a history of LABA/LAMA/ICS triple therapy and aimed to determine the effect of the long-term use of ICS prescriptions on the sputum microbial community structure and diversity based on metagenomic next-generation sequencing (mNGS). In addition, associations between microbial characteristics and clinical outcome-related airway function/biochemical indicators were further assessed in this study. The results provide a data reference to the rational and appropriate use of ICS and some elucidation of the role of sputum microbiota in the progression of COPD.

## 2 Materials and methods

### 2.1 Participants

A total of 60 stable COPD patients who had used ICS were recruited in the outpatient unit of the Respiratory Department, the Second Affiliated Hospital of China Medical University from December 2021 to September 2022. The diagnosis of COPD was based on the Global Initiative for COPD guidelines (Vestbo et al., 2013): The post-bronchodilator FEV1/FVC ratio was less than 70%. Participants aged 50–80 years who had maintained stable and received COPD drug therapy were enrolled in the study. All patients accepted and signed their informed consent to share their clinical data and undergo sputum sample collection. The procedure of the study was reviewed and approved by the Ethics Committee of the Second Affiliated Hospital of China Medical University (No. 2021PS815K).

The exclusion criteria for participants in this study were as follows: 1) COPD patients comorbid with a clinically active tumor, cardiac, hepatic, or kidney disease et al.; 2) COPD patients complicated with other acute or chronic lung disease, such as bronchiectasis, pulmonary tuberculosis, cystic fibrosis, pulmonary embolism, or pulmonary edema, et al.; 3) COPD patients who had taken microbial preparations, such as probiotics, prebiotics, or synbiotics; and 4) COPD patients who had received antibiotics, immunosuppressants, or cytotoxic drugs within 8 weeks.

### 2.2 Study design and clinical data collection

The study was a single-center, retrospective cohort study. The 60 recruited COPD patients were classified into two groups: the long-term ICS group and the short-term ICS group, based on the ICS medication administered within the second year prior (starting from 730 days prior up until 365 days prior to recruitment). COPD patients in the short-term ICS group were mainly treated with LABA and LAMA, with ICS added for no longer than 3 months; COPD patients in the long-term ICS group were treated with triple therapy (LABA, LAMA, and ICS) and had continuously used ICS for a period lasting more than 3 months in their prior regimen.

Demographic features of COPD patients were abstracted from electronic medical records. Pulmonary function, body temperature, and hemogram data were assessed at the time of enrollment, while sputum samples were collected for subsequent mNGS analyses. The course of the disease and pack-year were self-reported by the patients. COPD patients were also asked to recall the number of readmissions for acute exacerbation in the preceding year (starting from 365 days prior until the recruitment day), which was verified with medical records.

The primary outcome was the difference in the diversity of sputum microbial composition between the two groups. The secondary outcome included the difference in the relative abundance of the most common and potentially pathogenic sputum bacteria. Correlations between the sputum microbes and clinical characteristic indicators were also determined in the study.

### 2.3 Sputum sample collection

Induced sputum samples were collected on the day of enrollment. Prior to sputum collection, participants were requested to rinse their mouths with sterile saline thoroughly and to clear up their nasal and oral secretions for collection. All sputum samples were collected by the same physician while strictly following sterile operating procedures. Briefly, participants inhaled hypertonic saline (3%, 4%, and 5%, each for 10 min) for three sessions via an ultrasonic nebulizer; within 20 min, at least 4 mL of induced sputum was collected per patient into a sterile container. An aliquot (approximately 0.2 mL) of each sputum sample was taken for cell classification and counting. Sputum samples with a white blood cell count ≥25 and squamous cell count ≤10 under a low magnification field were considered to qualify. These qualified sputum samples were then divided into two tubes and stored at −80°C. Finally, the sputum samples were sent to Genoxor Medical Science and Technology Inc. to undergo metagenomic next-generation sequencing (mNGS) and follow-up bioinformatic analysis.

### 2.4 DNA extraction and mNGS

Approximately 0.2 mL of collected sputum per sample was resuspended in 1% DTT solution and proteinase K (20 mg·mL^-1^). The mixed liquids were then incubated at 56°C for 20 mins, followed by centrifugation to reduce the host nucleic acid background. The treated samples were then vortexed with enzyme and glass beads at 2800–3200 rpm for 30 min. At least 0.3 mL of the mixed liquid per sample was separated and extracted using a TIANamp Micro DNA Kit (DP316, Tiangen Biotech) according to the manufacturer’s instructions.

DNA libraries were constructed using an end-repair technique, followed by the addition of adapters, which were left overnight. Subsequently, PCR amplification was performed using an Ion Torrent Proton Sequencer (Life Technologies, Carlsbad, California). The quality of the DNA libraries was evaluated using an Agilent 2100 Bioanalyzer (Agilent Technologies, Santa Clara, California) combined with qPCR. Qualified DNA libraries were prepared using emulsion PCR in a OneTouch system. They were then sequenced on the NextSeq™ 550DX platform, SE-75 type.

The quality control of mNGS raw reads was conducted using Trimmomatic v0.36 to remove the low-quality tails, reads, and connector sequences. Subsequently, human reads were removed by mapping them to the human genome GRCh37, while microbial reads were retained and deposited in the database (SRA Num. PRJNA932550). In the PCR step, the FASTX-Toolkit, FastUniq, Fulcrum, and CD-HIT-DUP tools were used to delete duplicated reads. The taxonomic classification of microbial reads was undertaken by Kraken v2.0.9-beta and further estimated by the Bayesian algorithm. The relative expression of each species was computed using reads per kilobase of transcript per million mapped reads (RPKM) according to the following formula: gene reads/[the total mapped reads (millions) × genome length (KB)].

### 2.5 Bioinformatic analysis

The cumulative numbers of sputum microbiota at different taxonomic levels were analyzed using the *specaccum* function of the vegan package (Oksanen et al., 2015). A Venn diagram was generated using the *venn. diagram* function of the VennDiagram package. The alpha diversity at the phylum and genus levels were analyzed with the ACE, Chao, Sobs, Simpson, and Shannon indices using the *estimateR* function of the vegan package. For beta diversity analysis, samples were clustered using the hclust function of stats package (Tomita et al., 2016). Non-metric multidimensional scaling (NMDS) was created using the *metaMDS* function of the vegan package. Analysis of community similarities (ANOSIM) was performed using the *anosim* function. Partial least squares-discriminant analysis (PLS-DA) was performed using the *plsda* function of the mixOmic package (Fiehn, 2016). Linear discriminant analysis (LDA) effect size (LEfSe) analysis was conducted by the use of the Galaxy-based LEfSe tool; the LDA value was set at 2 and *p* < 0.05 was considered statistically significant (http://huttenhower.sph.harvard.edu/lefse/).

### 2.6 Statistical analysis

Sample size calculations were based on data obtained from a previous study by Contoli, M. et al. (Contoli et al., 2017) The mean difference of the Shannon index between the long-term ICS group and short-term ICS group was 0.244; the standard deviation between the two groups was 0.325 in the sputum samples of COPD patients. Considering an alpha of 0.05 and a power of 80%, the sample size of the study was 30 for each group (the calculated size was 28).

IBM Statistics SPSS 25.0 (IBM Corp, New York, United States), R (version 4.2.0), and GraphPad Prism 9 (GraphPad Software Inc., San Diego, United States) were used for statistical analyses and data plotting. Mann-Whitney U tests were used for continuous data; the Chi-square test was used for categorical variables. Spearman’s rank correlation analysis was used to analyze the correlation between microbial characteristics and clinical indices. All *p-*values were two-sided and those <0.05 were regarded as statistically significant.

## 3 Results

### 3.1 Basic information of participating COPD patients

A total of 60 COPD patients were included in the retrospective cohort of the study and classified into the short-term ICS group and long-term ICS group based on the duration of ICS treatment (whether or not the treatment lasted more than 3 months) in their prior treatment regimen within the year prior to the previous year (ICS duration short-term *versus* long-term: 2.83 ± 1.96 vs. 7.40 ± 2.28) (*p <* 0.05). The short-term group included 30 COPD patients with a mean age of 65.98 ± 7.11, of whom, 17/30 (56.67%) were males; the average course of the disease was 9.02 ± 3.35 years and the average number of pack-years was 28.84 ± 12.42. There were also 30 COPD patients in the long-term ICS group with a mean age of 65.22 ± 5.98, of whom, 18/30 (60.00%) were males; the average course of the disease was 9.46 ± 3.08 years and the average number of pack-years was 30.84 ± 15.66. There were no significant differences observed between the two groups in terms of age, sex, body temperature, course of disease, pack-years, or pulmonary function indicators (*p* > 0.05). The clinical information included the following: white blood cell count, neutrophil %, eosinophil %, and number of acute exacerbations and hospital admissions in the preceding year. As shown in Table 1, except for the neutrophil % and number of acute exacerbations and hospital admissions in the preceding year, the white blood cell counts and eosinophil % differed significantly between the two groups (*p* < 0.01).

**TABLE 1:** Demographic features and clinical information of participating COPD patients.

Variables	Short-term ICS (*n* = 30)	Long-term ICS (*n* = 30)	*p*-value
Age (mean ± SD)	65.98 ± 7.11	65.22 ± 5.98	0.653
Male Sex (n, %)	17 (56.67%)	18 (60.00%)	0.798
Body temperature (°C, mean ± SD)	36.53 ± 0.25	36.51 ± 0.22	0.702
Course of disease (year, mean ± SD)	9.02 ± 3.35	9.46 ± 3.08	0.601
Pack-years (mean ± SD)^a^	28.84 ± 12.42	30.84 ± 15.66	0.587
Pre FVC (L, mean ± SD)	3.40 ± 0.70	3.32 ± 0.74	0.690
Pre FVC (% of predicted)	61.14 ± 16.60	64.25 ± 17.73	0.485
Pre FEV1, L	2.69 ± 0.51	2.58 ± 0.63	0.471
Pre FEV1, (% of predicted)	39.01 ± 15.78	44.15 ± 19.91	0.272
Post FVC, L	2.26 ± 0.67	2.38 ± 0.83	0.536
Post FVC, (% of Post predicted)	67.53 ± 17.00	70.26 ± 16.99	0.537
Post FEV1, L	1.17 ± 0.48	1.30 ± 0.65	0.358
Post FEV1, (% of Post predicted)	44.72 ± 16.09	48.67 ± 19.89	0.401
White blood cell (×10^9^, mean ± SD)	6.44 ± 1.97	8.3 ± 2.08	0.001**
Neutrophil% (%, mean ± SD)	64.76 ± 11.70	69.93 ± 13.33	0.116
Eosinophil% (%, mean ± SD)	2.61 ± 1.17	6.77 ± 1.57	0.000**
ICS duration (months, mean ± SD)	2.83 ± 1.96	7.40 ± 2.28	0.037*
Number of acute exacerbations and hospital admissions (mean ± SD)	0.83 ± 0.84	1.00 ± 0.90	0.785

### 3.2 Effects of long-term ICS on the microbial composition and diversity of the sputum at the genus level

**FIGURE 1:**
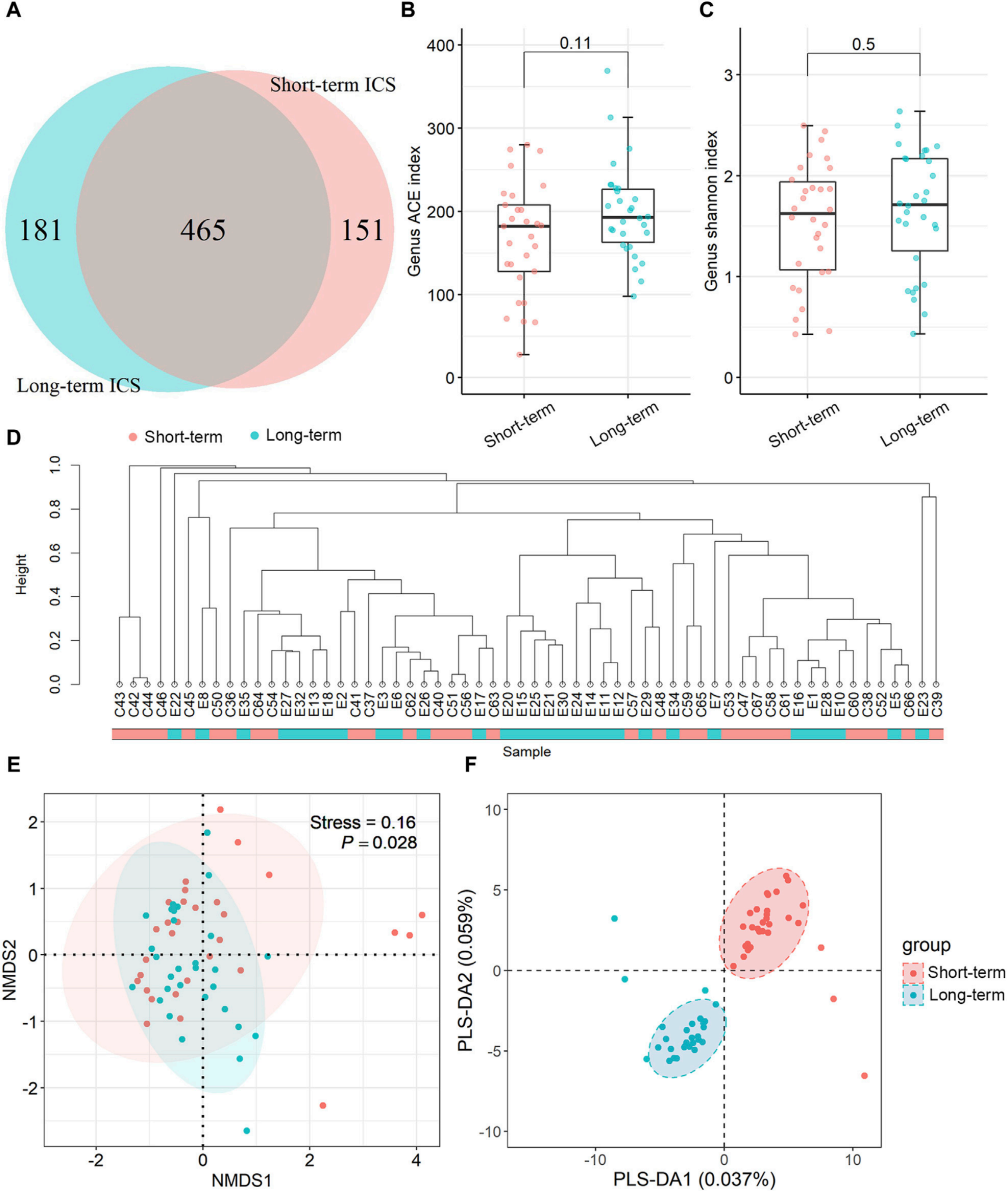
Microbial composition and diversity of sputum samples at the genus level based on metagenomic next-generation sequencing (mNGS). **(A)** Venn diagram of differential microbes. **(B,C)** Alpha diversity comparison based on the ACE index and Shannon index. Data in the boxplot are shown as the median and interquartile range. **(D)** Taxonomic tree visualization based on UPGMA clustering. **(E)** NMDS plot based on Bray-Curtis distance analysis. **(F)** Score plot of the PLS-DA model. Ellipses in **(E,F)** represent a 95% confidence level. N = 30 for each group.

As shown in the Supplementary Material (Supplementary Figure S1), the accumulation curve at the genus level tended to be smooth, indicating that the depth of mNGS was sufficient for further analysis. The Venn diagram results showed that 465 identified microbes were simultaneously detected in both of the two groups, while 151 and 181 microbes were present in the short-term and long-term ICS group, respectively (Figure 1A). The alpha diversity at the genus level was estimated by the ACE index and the Shannon index. Compared with the short-term group, the ACE index was increased in the long-term ICS group, but this difference was not statistically significant (Figure 1B). The Shannon index of the two groups showed no significant difference (Figure 1C).

Beta diversity was used to compare the community diversity between samples. Based on the UPGMA analysis, the taxonomic tree results showed that most of the samples were distributed in two clusters (Figure 1D). Differences in the community structure were analyzed using NMDS based on Bray-Curtis distance and the stress for NMDS was 0.16. As shown in Figure 1E, significant regional differences were found between the two groups (*p* < 0.05). Community similarity results (by ANOSIM analysis) showed that the between-group differences were greater than within-group differences (*p* < 0.05) (shown in the Supplementary Material, Supplementary Figure S2). The PLS-DA score plot displays the clustering results between the short-term and long-term ICS groups and the results in Figure 1F show substantial separation of the two groups.

### 3.3 Effects of long-term ICS on the relative abundance of sputum microbes at the phylum and genus levels

**FIGURE 2:**
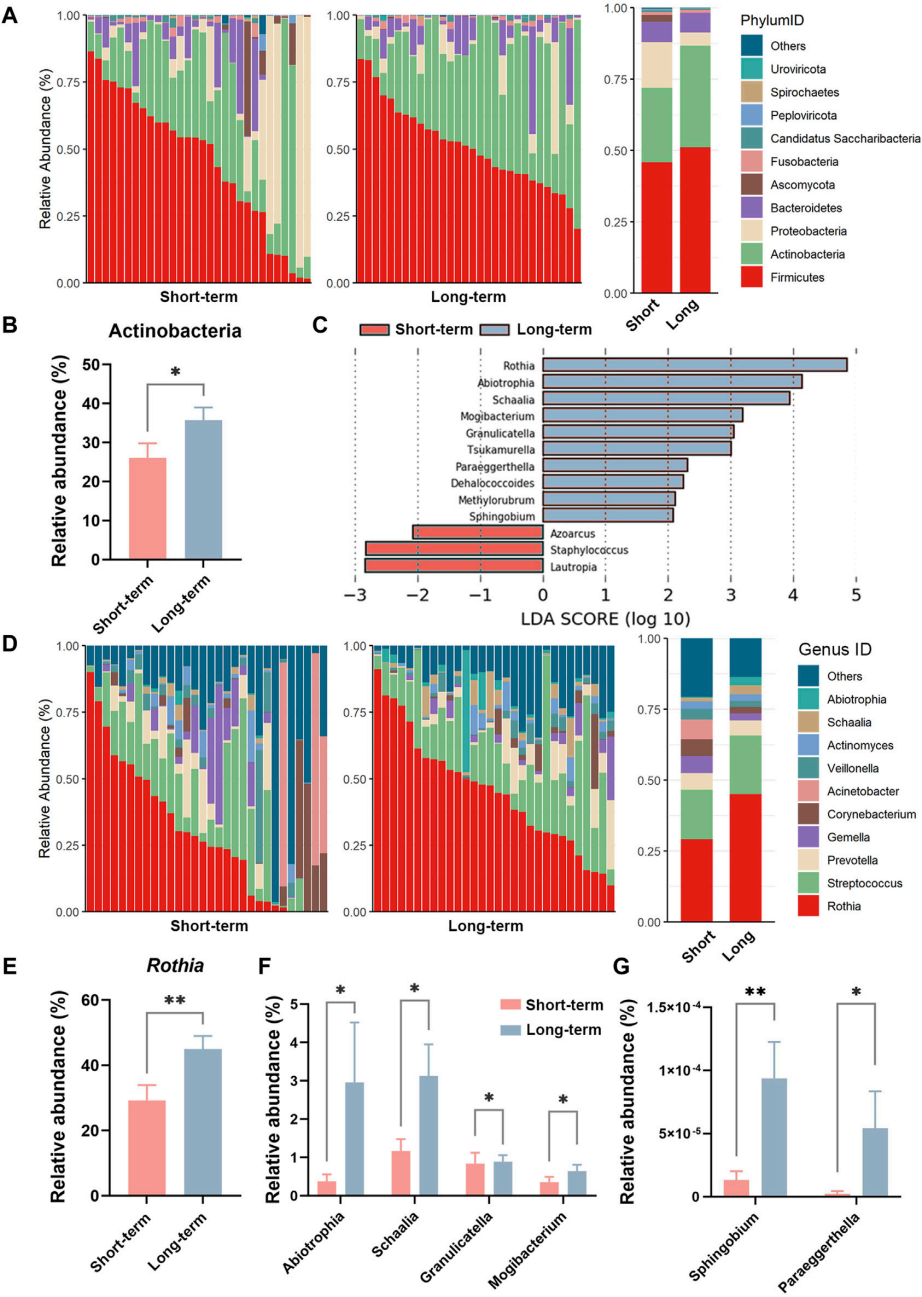
Relative abundance of sputum microbes at the phylum and genus levels based on mNGS. **(A)** Relative abundances of the top ten microbes at the phylum level. **(B)** Relative abundance of the Actinobacteria phylum. **(C)** LDA score of LEfSe analysis at the genus level (threshold >2.0). **(D)** Relative abundances of the top ten microbes at the genus level. **(E)** Relative abundance of the *Rothia* genus. **(F)** Relative abundances of the *Abiotrophia*, *Schaalia*, *Granulicatella*, and *Mogibacterium* genera. **(G)** Relative abundances of the *Sphingobium* and *Paraeggerthella* genera. Data are shown as mean ± SEM (N = 30 for each group). *p < 0.05; **p < 0.01.

As shown in Figure 2A, at the phylum level, the top three phyla in the sputum microbe were Firmicutes, Actinobacteria, and Proteobacteria in the short-term group, and these constituted approximately 88.00% of the total community. In the long-term ICS group, the top three members of the phylum were Firmicutes, Actinobacteria, and Bacteroidetes (91.41%). Compared with the short-term group, the relative abundance of the Actinobacteria phylum was significantly higher in the long-term ICS group (*p* < 0.05) (Figure 2B). At the genus level, *Rothia*, *Streptococcus*, *Acinetobacter*, *Gemella*, and *Corynebacterium* constituted the majority of the community in the short-term group (65.55%). In the long-term ICS group, *Rothia*, *Streptococcus*, *Prevotella*, *Schaalia*, and *Abiotrophia* constituted approximately 77.12% of the total community (Figure 2D). As shown in Figure 2C, we further screened the differential microbes among the genera using LEfSe analysis. At the genus level, the relative abundances of *Azoarcus*, *Staphylococcus*, and *Lautropia* in the short-term control group were significantly greater than that in the long-term ICS group (threshold >2.0). As shown in Figure 2E, the highest relative abundance of the *Rothia* genus was greater in the long-term ICS group compared with the control group (*p* < 0.01). Among the less abundant genera with relative abundances of >0.5%, the abundances of *Abiotrophia*, *Schaalia*, *Granulicatella*, and *Mogibacterium* were significantly higher in the long-term ICS group compared to the short-term ICS group (*p* < 0.05) (Figure 2F). As shown in Figure 2G, the relative abundances of the two genera *Sphingobium* (*p* < 0.01) and *Paraeggerthella* (*p* < 0.05), which are thought to be the potentially pathogenic microbes, were markedly higher in the long-term ICS group compared with short-term ICS group.

### 3.4 The relationship between sputum microbe alpha diversity and clinical parameters at the genus level

**FIGURE 3:**
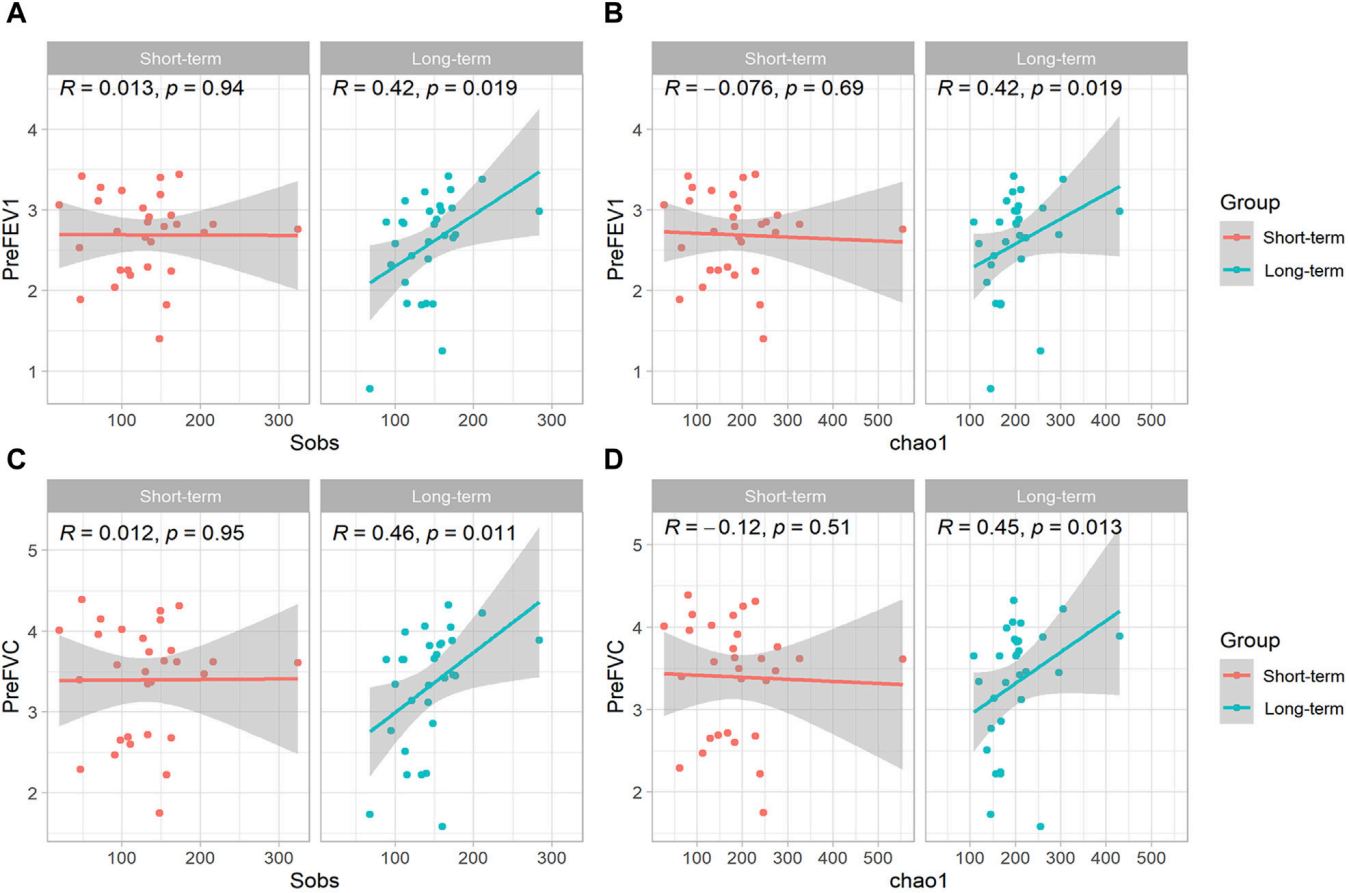
Scatterplot of the relationship between sputum microbe alpha diversity and COPD clinical parameters at the genus level. Scatterplot of the relationship between **(A)** Sobs index and Pre-FEV1; **(B)** Chao index and Pre-FEV1; **(C)** Sobs index and Pre-FVC; **(D)** Chao index and Pre-FVC. The solid curves were fitting curves; 95% confidence intervals are shown in gray. R-values represent correlation coefficients. The Spearman rank correlation coefficient was used for statistical analysis.

This study further analyzed the correlation between microbiota diversity at the genus level and clinical parameters. As shown in Figures 3A, B, the alpha diversity index (Sobs and Chao) of sputum microbes was positively correlated with the patients pre-bronchodilatory FEV1 (Pre-FEV1) in the long-term ICS group (R = 0.42, *p* < 0.05). No such significant correlation was found in the short-term control group. Similar positive associations were observed for the alpha diversity index of sputum microbes and pre-bronchodilatory FVC (Pre-FVC), as shown in Figures 3C, D (Sobs: R = 0.46, *p* < 0.01 and Chao: R = 0.45, *p* < 0.01).

### 3.5 Relationship between the relative abundance of sputum microbes and eosinophil % at the genus level

**FIGURE 4:**
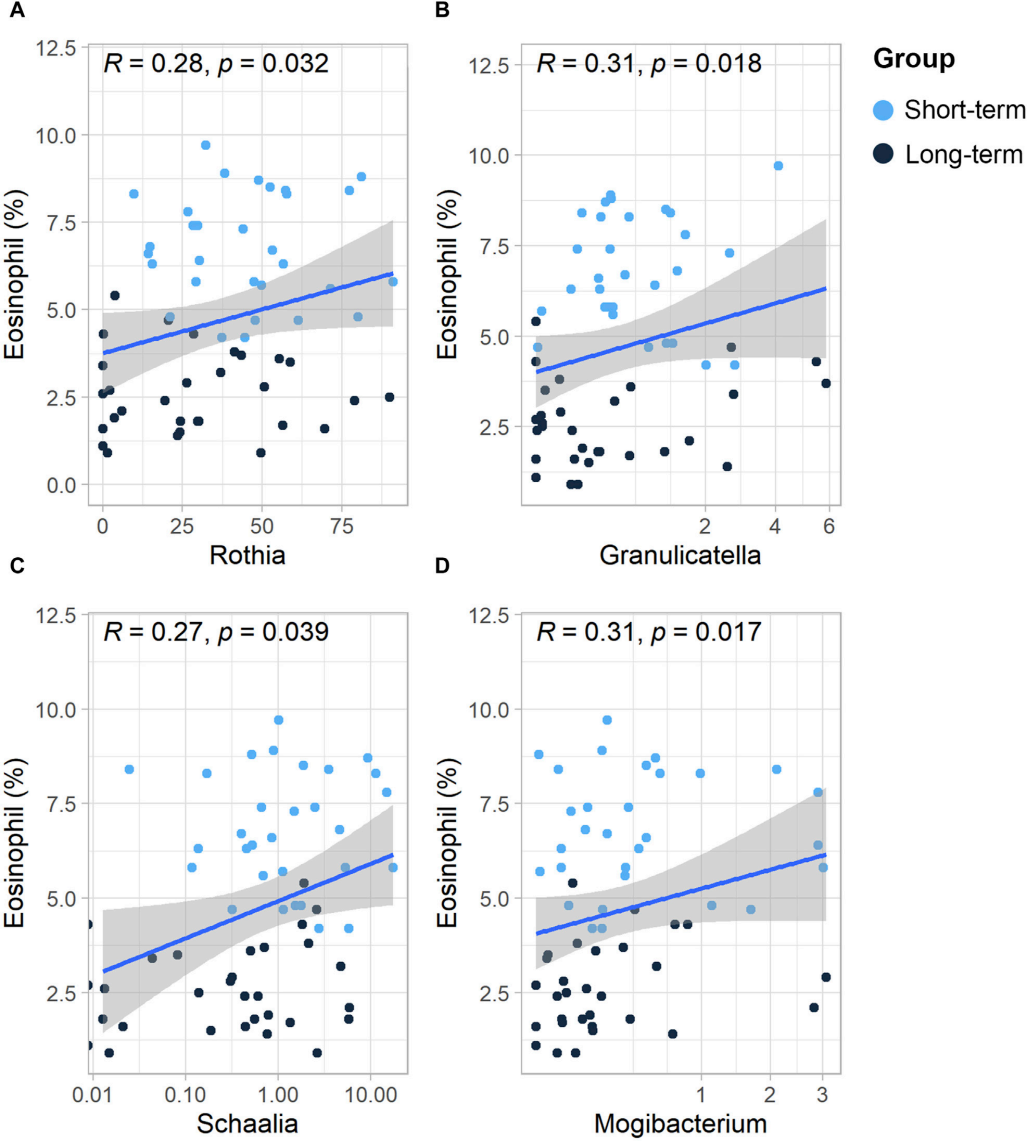
Scatterplot of the relationship between the relative abundance of sputum microbes and eosinophils (%) at the genus level. Scatterplot of the relationship between eosinophils (%) and the **(A)** *Rothia* genus, **(B)** *Granulicatella* genus, **(C)** *Sshaalia* genus, **(D)** and *Mogibacterium* genus. The solid curves are fitting curves; 95% confidence intervals are shown in gray. R-values represent correlation coefficients. The Spearman rank correlation coefficient was used for statistical analysis.

As shown in the Supplementary Material (Supplementary Figure S3), the eosinophil % in COPD patients was positively associated with the number of acute exacerbations and hospital admissions in the preceding year (*p* < 0.05). Therefore, we further analyzed the relationship between the relative abundance of sputum microbes and the eosinophil %. At the genus level, the relative abundances of the genera *Rothia*, *Granulicatella*, *Schaalia*, and *Mogibacterium* were all positively and significantly associated with the eosinophil % (*p* < 0.05) (Figures 4A–D).

## 4 Discussion

For the clinical outcome, this study mainly demonstrated that the long-term use of ICS-added triple therapy does not greatly contribute to clinical airway function or further decrease the number of acute exacerbations or hospital admissions when compared with short-term use. In terms of sputum microbiota, the long-term use of ICS significantly modified the community structure (beta diversity) of sputum microbiota among COPD patients. At the genus level, the relative abundances of *Rothia*, *Granulicatella*, *Schaalia*, *Mogibacterium, Sphingobium,* and *Paraeggerthella* were higher in the long-term ICS treatment group compared with the short-term ICS treatment group. The alpha diversity of sputum microbiota in the long-term ICS group was positively associated with the clinical pre-FEV1 and pre-FVC. In addition, the relative abundances of *Rothia*, *Granulicatella*, *Schaalia*, and *Mogibacterium* were all positively associated with the eosinophil % of COPD patients.

Alpha diversity indices are widely used for evaluating the richness and diversity of microbial communities. In a prospective study, Contoli et al. demonstrated that compared with a LABA-only treatment group 12 months’ of ICS/LABA treatment increased the Shannon index of COPD patients (Contoli et al., 2017), while Filho et al. reported that the richness and Shannon index were decreased after 12 weeks’ ICS/LABA treatment (Leitao Filho et al., 2021). In research by Pragman and Ramsheh et al., no significant difference was found in the alpha diversity between the ICS and control group (Pragman et al., 2019; Ramsheh et al., 2021). From these previous studies, the effects of ICS treatment on the alpha diversity of sputum microbiota were contradictory, and the duration of ICS use seems to be the reason for this inconsistency. Considering that the severity of COPD is usually more imbalanced in no-ICS and ICS-treated COPD patients (GOLD, 2023), we compared changes in the microbiota diversity between long-term and short-term ICS-treated patients, finding no significant difference in the alpha diversity of the sputum microbiota between the groups in the present study. Furthermore, similar to the results reported by a previous study by Contoli et al. involving 12 months’ of ICS/LABA treatment (Contoli et al., 2017), this study showed that the long-term use of ICS as part of triple therapy had the tendency to increase the alpha diversity of the microbiota at the genus level. Furthermore, ICS has been shown to coincide with beta diversity (Pragman et al., 2012), and the present study showed a substantial difference in the microbiota communities between the long-term and short-term ICS groups using multiple analysis methods for beta diversity. The above results support the view that different ICS formulations induce differential airway microbiome responses, which may further affect the clinical outcome of COPD patients (Leitao Filho et al., 2021).

The relative abundances of the phylum Actinobacteria and genus *Rothia* were significantly increased after long-term ICS treatment compared with short-term use. As a member of the Actinobacteria phylum, *Rothia* is a gram-positive cocci that is an opportunistic pathogen, causing immune diseases by producing enterobactin, particularly in those with compromised immune systems (Cai et al., 2022; Jeong et al., 2023). *Rothia* always reside in the oral cavity and pharynx. Its presence in the sputum could either be due to its migration from the mouth to the respiratory system (Rafiqul Islam et al., 2022) or long-term ICS-induced changes to the lung environment and various immunological factors. As one previous study reported, a higher level of Actinobacteria was found in AECOPD patients compared to those with stable type COPD, and this was associated with the genus *Rothia* (Su et al., 2022), indicating that the observed increase in the relative abundance of Actinobacteria might be associated with the acute exacerbation of COPD and promotes the higher ratio of eosinophils (% of white blood cells) among COPD patients.

At the genus level, a previous study demonstrated that COPD patients had higher relative abundances of *Abiotrophia* and *Mogibacterium versus* normal controls (Liu et al., 2023). The study further showed that long-term ICS use induced higher relative abundances of the genera *Abiotrophia*, *Schaalia*, *Granulicatella*, *Mogibacterium, Sphingobium,* and *Paraeggerthella* when compared with the short-term ICS group. As opportunistic pathogens, *Abiotrophia* and *Granulicatella* are associated with various clinically relevant infections (Zheng et al., 2004; Hosgood et al., 2014). The increase in the relative abundance of the *Granulicatella* genus in particular has been linked to patient sputum eosinophilia (Wang et al., 2021), which may indicate a higher risk of acute exacerbations in COPD. Furthermore, as a gram-positive genus of bacteria, *Mogibacterium* has been reported to be associated with persistent generalized disease (Liu et al., 2023) and acute lung infections (Hill et al., 1987). The above may indicate that the long-term use of ICS-added triple therapy cannot further decrease the increasing relative abundance of microbiota associated with COPD when compared with the short-term treatment group, even resulting in the opposite outcome.

Pre-FEV1 and Pre-FVC are considered objective measures of respiratory health (Zhang et al., 2023). As previous studies have demonstrated, reduced microbiota alpha diversity is associated with a worsening of FEV1 and an increased risk of COPD exacerbation (Erb-Downward et al., 2011; Han et al., 2012). In addition, a positive association has been found between decreased microbiota alpha diversity and FEV1 decline in cystic fibrosis and other diseases characterized by the obstruction of the airway (Soret et al., 2020). In the current study, similar positive associations were observed for alpha diversity indices (Sobs and Chao) of sputum microbes and clinical Pre-FEV1 and Pre-FVC in the long-term ICS group; however, no such relationship was found in the short-term ICS group. We suspect that the reason for these inconsistent results may be related to alterations in the community structure of sputum microbiota between the two groups: the long-term use of ICS might decrease the relative abundance of pro-inflammatory microbiota while increasing the relative abundance of anti-inflammatory microbiota, though the difference in alpha diversity was not found to be significant. This hypothesis awaits longitudinal follow-up studies.

Eosinophils are involved in inflammatory reactions and participate in immune modulation. A higher eosinophil % (of all white blood cells) indicates a severe level of COPD disease (Kämpe et al., 2012). The bacterial load has a positive association with eosinophil counts in COPD patients (Kolsum et al., 2017; Müllerová et al., 2019). The present study found that the relative abundances of the genera *Rothia*, *Granulicatella*, *Schaalia*, and *Mogibacterium* were positively associated with the eosinophil % in ICS-treated COPD patients, which means that the increase in the relative abundances of these genera may promote the inflammatory reaction during the period of treatment with ICS-included triple therapy. This finding provides potential intervening targets in the microflora, which may be considered in the design of follow-up studies. Researchers may consider adding the above genera to *in vivo* and *in vitro* systems in order to ascertain the mechanism by which the microbiota influences inflammation and immune reactions. Such acquired data might then be used to construct a reference index to better determine the prognosis of COPD patients.

The limited sample size means the results of the study can only be considered as preliminary, and as a retrospective cohort study, this study includes all limitations inherent to such studies. Furthermore, the patients’ reported ICS dosage may have been inaccurate, and this factor was not assessed in the present study. The influence of the ICS dose on the microbiota remains to be further investigated. Therefore, further research should contemplate implementing a multicenter, prospective, large sample size design in order to provide more credible data, rather than relying on patients’ retrospective recall. Another limitation was that the samples used in the study were collected from the lower airway, so microbiota in the sputum samples were at risk having been contaminated by the upper respiratory tract. Finally, normal control COPD patients (no ICS treatment) were not included in the study, and the total white blood cell counts and eosinophil % (of all white blood cells) were not equal between the two groups. Despite these limitations, to our knowledge, the study is the first to compare alterations in sputum microbiota between long-term and short-term ICS-treated COPD patients.

## 5 Conclusion

In summary, the present study has found that compared with short-term ICS use, long-term ICS use could not further improve airway function or decrease the number of hospital admissions due to acute exacerbation. The long-term use of ICS altered the beta diversity of sputum microbiota among COPD patients compared with short-term use. In the long-term ICS group, the alpha diversity of sputum microbiota was positively associated with clinical Pre-FEV1 and Pre-FVC. In addition, the relative abundances of *Rothia*, *Granulicatella*, *Schaalia*, and *Mogibacterium* may associated with the clinical outcome of COPD patients. The findings of this study identify characteristic sputum microbiota profiles resulting from short-term and long-term ICS use in COPD patients and demonstrate an association between the microbiota structure and airway functional indicators, which may help optimize the clinical decision-making of ICS use in COPD patients.

## Data Availability

The datasets presented in this study can be found in NCBI, BioProject, Accession: PRJNA1050019, available at https://www.ncbi.nlm.nih.gov/bioproject/PRJNA1050019.
