# Supplementary material for: Sputum microbe community alterations induced by long-term inhaled corticosteroid use are associated with airway function in chronic obstructive pulmonary disease patients based on metagenomic next-generation sequencing (mNGS)
Source: Front Pharmacol. 2024 Jun 10;15:1323613. doi: 10.3389/fphar.2024.1323613 (PMC11194361; doi:10.3389/fphar.2024.1323613)
Supplement: Supplementary file 1 [file DataSheet1.docx]

Supplementary Material

# Supplementary Figures

## Supplementary Figure 1
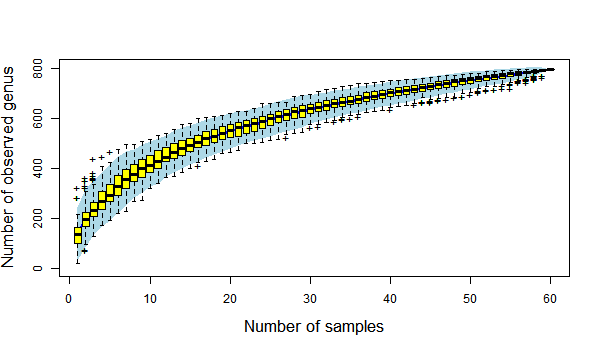


**Supplementary Figure 1.** Microbial accumulation curve of sputum samples at the genus level based on metagenomic next-generation sequencing (mNGS). The accumulation curve at the genus level tended to be smooth, indicating that the depth of mNGS was sufficient for further analysis.

## Supplementary Figure 2

##
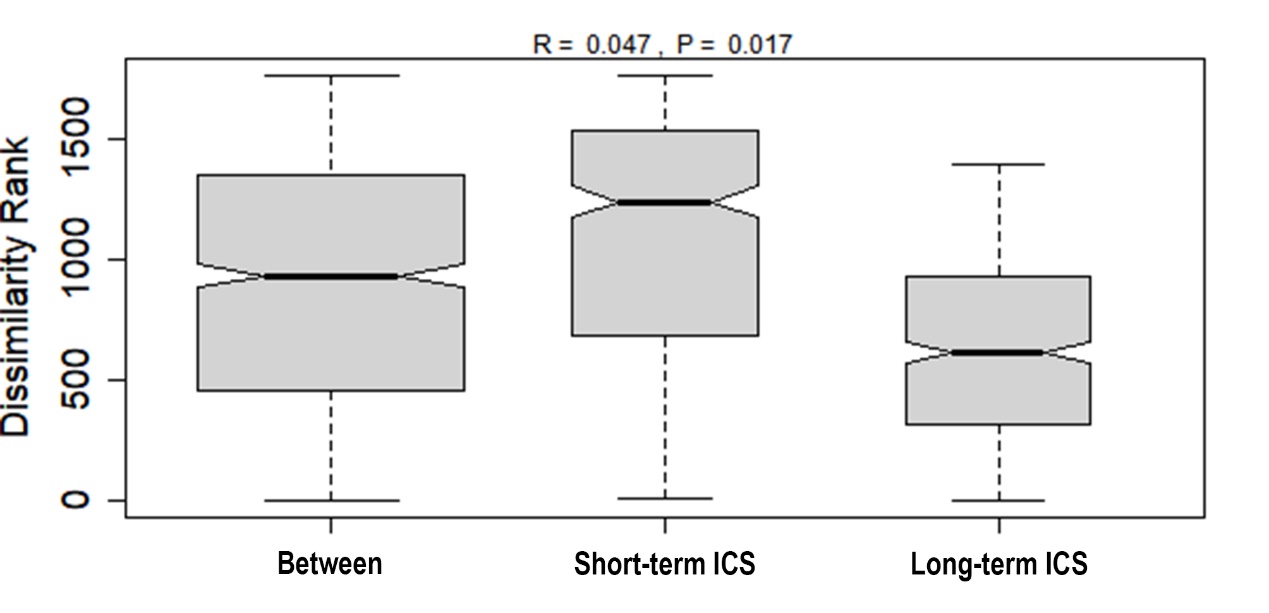


**Supplementary Figure 2.** Similarity of bacteria community of sputum samples among COPD patients. Data in the boxplot are shown as the median and interquartile range. The result shown that the between-group differences were greater than within-group differences.

## Supplementary Figure 3

##
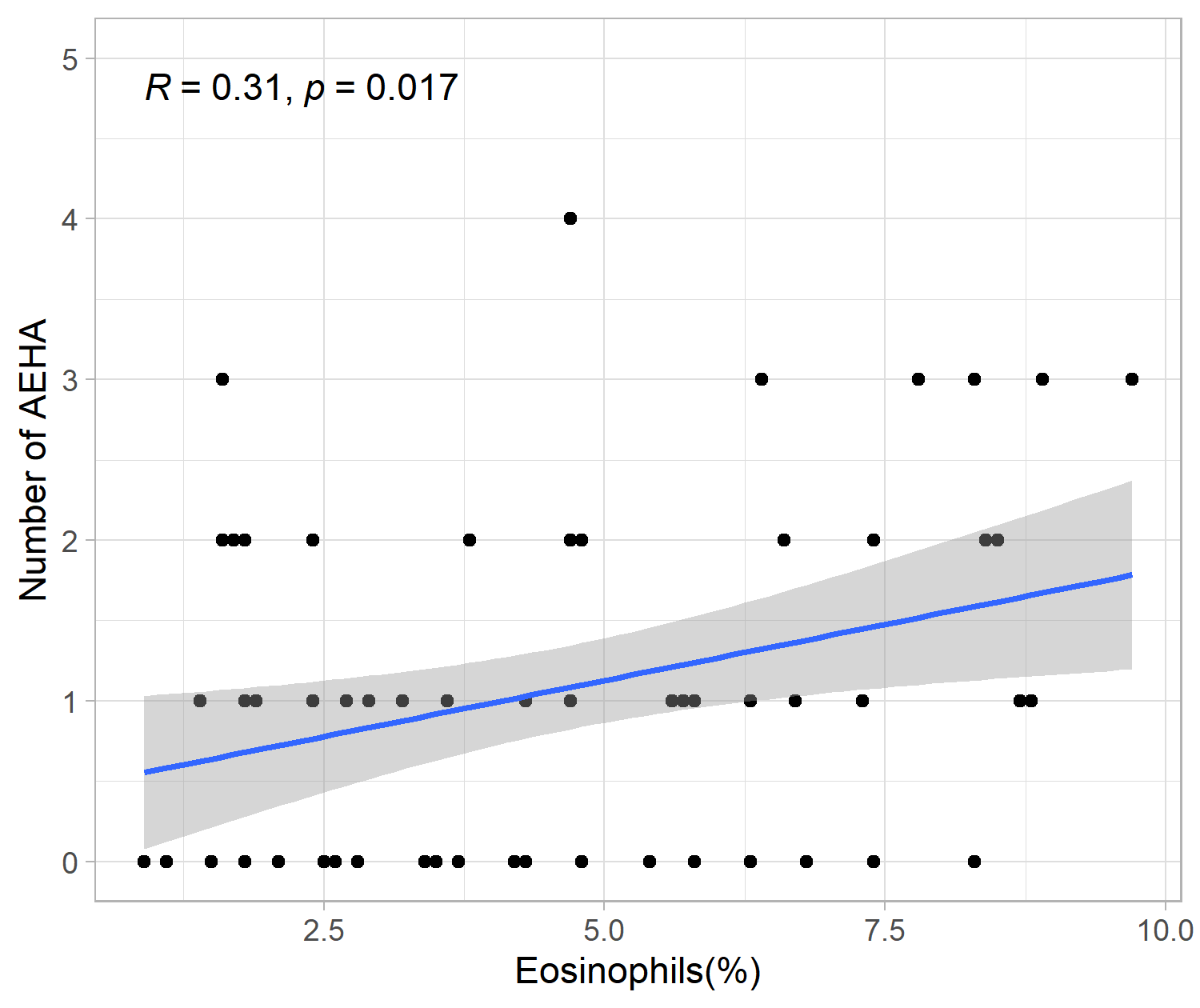


**Supplementary Figure 3.** Scatterplot of the relationship between eosinophils (%) and the number of acute exacerbations and hospital admissions (AEHA). As shown in the Fig. S3, the eosinophil % in COPD patients was positively associated with the number of acute exacerbations and hospital admissions in the preceding year (*P* < 0.05).
